# Fracture Risk Evaluation of Bone Metastases: A Burning Issue

**DOI:** 10.3390/cancers13225711

**Published:** 2021-11-15

**Authors:** Cyrille B. Confavreux, Helene Follet, David Mitton, Jean Baptiste Pialat, Philippe Clézardin

**Affiliations:** 1 Centre Expert des Métastases Osseuses (CEMOS), Département de Rhumatologie, Institut de Cancérologie des Hospices Civils de Lyon (IC-HCL), Hôpital Lyon Sud, Hospices Civils de Lyon, 69310 Pierre Bénite, France; 2 Université de Lyon, Université Claude Bernard Lyon 1, 69100 Villeurbanne, France; helene.follet@inserm.fr (H.F.); jean-baptiste.pialat@chu-lyon.fr (J.B.P.); philippe.clezardin@inserm.fr (P.C.); 3 Institut National de la Santé et de la Recherche Médicale INSERM, LYOS UMR1033, 69008 Lyon, France; 4 Université de Lyon, Université Gustave Eiffel, Université Claude Bernard Lyon 1, LBMC, UMR_T 9406, 69622 Lyon, France; david.mitton@univ-eiffel.fr; 5 CREATIS, CNRS UMR 5220, INSERM U1294, INSA Lyon, Université Jean Monnet Saint-Etienne, 42000 Saint-Etienne, France; 6 Service de Radiologie, Centre Hospitalier Lyon Sud, Hospices Civils de Lyon, 69310 Pierre Bénite, France

**Keywords:** bone metastasis, pathological fracture, mirels’ score, spinal instability, neoplastic score, finite element analysis

## Abstract

**Simple Summary:**

Major progress has been achieved in stage-IV bone metastatic patients to control over the disease progression, thereby resulting in longer survival. Self-autonomy and return to physical activity are now frequent. Thus, assessment of the strength of tumoral bone has becoming an issue, especially with the rapid variations of bone tumoral aspect (from lytic to sclerosing and vice versa), that we can observe on treatment. This review will explain the current available imaging techniques, the limits of the existing fracture risk scores in bone metastasis and the new numerical simulation technics arising in biomechanics.

**Abstract:**

Major progress has been achieved to treat cancer patients and survival has improved considerably, even for stage-IV bone metastatic patients. Locomotive health has become a crucial issue for patient autonomy and quality of life. The centerpiece of the reflection lies in the fracture risk evaluation of bone metastasis to guide physician decision regarding physical activity, antiresorptive agent prescription, and local intervention by radiotherapy, surgery, and interventional radiology. A key mandatory step, since bone metastases may be asymptomatic and disseminated throughout the skeleton, is to identify the bone metastasis location by cartography, especially within weight-bearing bones. For every location, the fracture risk evaluation relies on qualitative approaches using imagery and scores such as Mirels and spinal instability neoplastic score (SINS). This approach, however, has important limitations and there is a need to develop new tools for bone metastatic and myeloma fracture risk evaluation. Personalized numerical simulation qCT-based imaging constitutes one of these emerging tools to assess bone tumoral strength and estimate the femoral and vertebral fracture risk. The next generation of numerical simulation and artificial intelligence will take into account multiple loadings to integrate movement and obtain conditions even closer to real-life, in order to guide patient rehabilitation and activity within a personalized-medicine approach.

## 1. Introduction

Bones constitute one of the most common sites for metastasis. The clinical cases submitted to our weekly bone metastasis multidisciplinary meetings highlight the benefits and challenges of modern therapies in all oncological specialties. For instance, novel treatment approaches have considerably enhanced the control over the disease progression, thereby resulting in longer survival; thus, self-autonomy, locomotion, and return to sport activities are becoming an issue. Moreover, locally, we now observe rapid variations of the bone metastasis aspect (from lytic to sclerosing) with some drugs, suggesting some major variability in the local bone strength. Currently, physicians are unable to precisely estimate this strength, and therefore to make recommendations. We thus observe an increasing gap between the existing fracture bone scoring classifications and the current bone metastatic patient local and global prognosis enabled by new targeted therapies. This topic is all the more important since these rapid bone changes may modify the care strategy, for example, choosing a temporary contention instead of orthopedic surgery, or opting for an aggressive eradicating treatment, such as stereotaxic radiotherapy on a regressive lesion initially considered as too large but related to potential neurological complications after relapse. Thus, there is a need to develop new tools to reliably estimate the bone metastatic strength and its progression in order to optimize patient care strategies and mid- and long-term physical activity. Moreover, these bone metastasis meetings deal with both long bones and vertebrae, hence these tools should be usable for both locations and, in the future, include the strains related to movements and sport activities. In addition, the bone strength behavior of solid bone metastasis and myeloma bone lesions is probably different and deserves a separated analysis. All these points needed to be reviewed, from bedside to bench, in a single manuscript gathering all research fields and considering both bone locations (vertebrae and long bones). This challenge could be taken up with the contribution of a unique consortium, allowing a multidisciplinary approach, consisting of a bone metastasis physician (CBC), an osteoarticular radiologist (JBP), and fundamental expert research scientists in the pathophysiology of bone metastasis (PC) and bone biomechanics and numerical simulation (HF and DM). This group already works together on a daily basis on the MEKANOS research protocol and thus offers a holistic top-level translational approach to serve patients. Interestingly, the progress made by the consortium has led to a new care strategy and conceptualization, as presented in the last figure of the review.

## 2. Main Pathophysiological and Clinical Features of Bone Metastases

Bone dissemination occurs early during cancer progression and follows a complex multistep process, involving specific cellular properties of some cancer cell sub-populations of the primary tumor, such as loss of cell-cell interactions, epithelial-to-mesenchymal transition, migration/invasion, and dissemination through circulation [1,2,3,4,5]. These properties are required for cancer cells to colonize a distant organ, settle in this new environment, and create a metastatic niche. In the bone, the pattern of metastatic spread follows the distribution of the red bone marrow, such as in vertebrae, sternum, pelvis, and epiphysis/metaphysis of long bones, where the vascularization and hematopoiesis are enriched. Once in the bone marrow niche, these cells, known as disseminated tumor cells (DTC), may remain quiescent, sometimes for years or decades, before they become clinically detectable [6]. This period of latency is known as tumor dormancy and involves a dynamic interplay between cancer cells and cells from the bone marrow microenvironment, such as spindle-shaped N-cadherin+/CD45- osteoblast (SNO) cells, CXCL-12-abundant reticular (CAR) cells, stromal cells, mesenchymal stem cells, and immune cells [4,7,8,9]. Changes in the bone environment in favor of osteoclast-mediated bone resorption are sufficient to trigger dormant cell reactivation, which is why bone-targeted agents, such as bisphosphonates, by decreasing bone resorption, improve the elimination of DTCs in the bone marrow of breast cancer patients with a minimal residual disease [4,10]. Additional signals are likely to be involved in tumor cell reactivation, and the identification of these molecular players is under intense investigation as they might represent an opportunity for therapeutic targeting. Upon reactivation, tumor cells proliferate and alter the functions of osteoclasts and osteoblasts, promoting skeletal destruction. Insights into the molecular mechanisms that either initiate, promote, or both, the development of bone metastases have recently been extensively reviewed [4,11].

Because tumor cells disseminate in the bone marrow long before the development of clinically detectable metastases, and dormant cell reactivation leads to the development of bone metastases that remain localized or widespread throughout the skeleton [4], it is essential in the clinic to conduct a comprehensive screening of the skeleton for the presence of bone (micro)metastases using positron emission tomography (PET)-scan or magnetic resonance imaging (MRI). However, due to the imaging resolution limit (about 3–5 mm), the presence of DTCs can never be ruled out. Bone metastases can be asymptomatic. The first symptom occurring in patients with symptomatic bone metastases is most often a continuous, stubborn, rising bone pain that requires simple to morphine pain killers. At some stages of the bone disease, pain can be so severe that local actions such as radiotherapy, surgery, or interventional radiology are necessary. In parallel with bone pain, symptomatic bone metastases expose patients to secondary fractures and nerve or medullary compression. It is estimated that 50% of progressing bone metastases will lead to skeletal complications in the absence of a treatment to reduce bone resorption [12,13,14]. Rosen et al. also found that skeletal complications occur within a short period of time (6 months) after diagnosis, indicating that an active treatment inhibiting bone resorption should not be delayed [15]. Moreover, bone metastasis complications worsen the quality of life, delay cancer treatments, and increase mortality [16]. A localized fracture occurring on a cancer lesion is much more complicated to treat than a non-fractured metastatic lesion preplanned for surgical intervention. It is common to observe a delayed consolidation or even a pseudarthrosis. Thus, oncologists ought to develop transversal multidisciplinary health care teams involving onco-rheumatologists, cancer trained orthopedic surgeons, locomotor radiologists, radiation therapists, and the referral oncologist. These health care teams would work to prevent skeletal-related events and pursue three goals: upstream assessment of pathological fracture risk, bone resorption inhibitor indications, and collegial indications of local interventions, taking into account pain, fracture risk, cancer drugs, cancer progression, and patient prognosis, general conditions, and wishes [17]. Trying to avoid making decisions in a hurry at the emergency unit, and on the contrary, promoting pre-planned well-balanced decisions is essential, and all the more important since cancer surgery regularly requires specific techniques and skills [18] that are not always accessible in emergency settings. This strategy optimizes patient health and survival and encourages initiatives to develop multidisciplinary bone oncology consultation meetings [17].

## 3. Bone Metastases: New Clinical Insights

The epidemiology of bone metastasis was established in the late 1990s by Coleman and Rubens [19,20]. Bone metastases are frequent complications of several cancers, including breast and prostate cancer, for which the bone metastatic incidence is the highest (68–73%), and other osteophilic tumors such as lung, thyroid, kidney, and bladder cancer. Bone metastases, according to their radiological aspect, are mainly osteoblastic or osteolytic in prostate and lung cancer, respectively, or a mix of lytic and blastic lesions in other osteophilic cancer types [21]. From this epidemiological basis, three challenges have recently emerged.

(1) A personalized medicine based on the molecular diagnosis of the tumor. Molecular diagnosis of the tumor has enabled refining of the histological classification and has revealed considerable variations of overall survival among molecular subgroups. For instance, V-Ki-ras2 Kirsten rat sarcoma viral oncogene homolog (KRAS)-mutated adenocarcinoma lung cancer are associated with a poorer prognosis than wild type adenocarcinoma [22]. Variations within the histological type have also been observed for bone affinity; for example, Epidermal Growth Factor Receptor (EGFR)-mutated lung adenocarcinoma have a higher bone affinity than the one with ALK translocation [23,24,25,26]. Tumor molecular diagnosis used to be restricted to primary tumors and soft metastases, and is now routinely available for bone metastases [27].
(2) The advent of targeted therapy and immunotherapy have provoked a considerable increase in life expectancy, even for patients whose cancers have spread to distant parts of the body (stage IV). For example, gefitinib in lung cancer has drastically improved life expectancy [28]. Similarly, pembrolizumab has also improved life expectancy in lung cancer [29], even in stage IV metastatic cancers. Both these examples highlight that prognosis is prolonged far beyond the historical prognosis of synchronous bone metastatic lung adenocarcinoma [30]. Thus, more and more patients stabilize for a long period of time, which raises new questions about profit and loss balance for anti-resorptive agents and dose-intensity treatments. Indeed, bone metastatic patients in anti-resorptive agent phase III trials were treated during 24 months, however long-term data are still not available, while this clinical situation is becoming common. Furthermore, de-escalation studies are ongoing. Bisphosphonate studies have shown that after an initial monthly regimen, it is possible to space out the injections [31,32,33,34]. Data about denosumab, a monoclonal antibody and not a pyrophosphate analogue, are very scarce. Moreover, it is already known that soon after denosumab suspension, a bone remodeling flare occurs; this flare is conceptually not desirable for patients as it exposes them to a benign fracture cascade [35,36,37], highlighting the importance of blocking bone remodeling at the end of denosumab sequence using a powerful bisphosphonate. Interestingly, recent ESMO guidelines have evolved and propose a first switch toward a personalized bone antiresorptive agent prescription after an initial phase of 3–6 months of dose-dense monthly infusions [38].
(3) The observation of the high lability (transition from lytic to sclerotic aspect) of bone metastases with the use of targeted therapies. Indeed, it is amazing to observe how quickly a highly osteolytic lesion responding well to anti-hormonal treatment or to targeted therapies such as EGFR inhibitor treatment, may condense, within a short period of time [39]. A synergistic effect has also been observed in combination with Rankl inhibition [40].
(4) Bone turnover biomarkers potentially provide important insight for predicting the risk of disease relapse in cancer patients and for monitoring the response to antiresorptive therapies [4,41].

Altogether, these new clinical insights place the accuracy of the fracture risk assessment and its re-evaluation at the heart of the reflection. It nowadays appears as a key issue to guide locomotor care in bone metastatic patients through local treatments and personalized prescription of anti-resorptive agents. We will now describe the currently available fracture risk assessment methods and the emerging techniques that are under development.

## 4. Current Fracture Risk Evaluation of the Tumoral Bone

As multiple and severe complications are associated with bone metastatic fracture, early and accurate fracture risk evaluation of bone metastasis is a crucial issue. To achieve this goal, on the one hand, actions of education and awareness about bone oncology and bone metabolism should be aimed toward the oncological community. On the other hand, bone and cancer specialists should provide clear guidelines to prevent fracture onset. Since many bone metastases initially remain asymptomatic, the first step is to perform a comprehensive screening of bone metastasis locations. Every bone metastatic location should then be checked for pain, fracture, and nerve compression risk in order to propose a personalized strategy consistent with the general cancer treatment [17].

### 4.1. Bone Metastasis Cartography

The aim of the cartography is to obtain a comprehensive evaluation of bone metastasis locations, including long bearing bones, independently of their clinical features. This cartography should make a compromise between the time-consuming whole skeleton imaging and the frequency of bone metastatic location; thus, covering whole femurs and knees looks reasonable. This is not always routinely performed and many examinations cover only proximal femurs, which represents a lowering of chance in terms of fracture risk evaluation. Two imaging techniques dominate the cartography: bone scan and PET (positron emission tomography) scan.

Bone scan is the oldest available method to explore the whole skeleton, and relies on a radioactive labelled bisphosphonate that dispenses a low level of radiation (around 4 mSv). Bone scan detects bone metastases from 2 to 18 months earlier than plain radiographs, since a 5% bone involvement is sufficient to be detected. In published studies, the reported sensitivity and specificity vary between 62 and 100% and 61 and 100%, respectively. Bone scan sensitivity is high for sclerosing bone metastases such as the ones from prostate cancer. Bone scan performance has been improved up to a sensitivity and specificity of 98% and 81%, respectively, by coupling a low dose CT-scan (SPECT-CT). SPECT-CT allows the exploration of specific locations identified by the general body scanning. By contrast, its specificity remains poor for osteolytic lesions, such as in multiple myeloma, thyroid, kidney, and lung cancers. False negative results essentially correspond to very aggressive osteolytic lesions with a complete sideration of bone formation where bisphosphonates fix. In addition, bone scan does not provide any information about soft tissues [42]. PET-scan also belongs to the nuclear imaging category and combines the injection of a radioactive tracer with a coupled CT-scan to merge functional activity and anatomical locations [42,43]; 18-FDG is the most common available radioactive tracer. After injection, it is better captured by cells with a high metabolism, including cancer cells, but not exclusively. Hypermetabolism will also be observed in fracture, infection, or inflammation sites, leading to false positive results that should be clinically identified. By contrast, prostate cancer cells poorly uptake 18-FDG and generate false negative results. Therefore, choline radio-tracer is used for prostate cancer. Interestingly NaF probably has the highest sensitivity to detect bone metastases but its use is very restricted in many countries, and it does not provide soft tissue information. Once the cartography is performed, a targeted evaluation should be carried out for bone lesions, even asymptomatic, to determine the bone involvement and evaluate the fracture risk.

### 4.2. Local Evaluation of Bone Metastasis

Radiographs—Plain radiographs are easy to perform, accessible, and allow a rapid evaluation of the whole skeleton, but have a limited sensitivity because of superimpositions and because significant bone lysis is necessary. Indeed, almost 50% of the trabecular bone has to at least be impaired for lesions to be detected [44]. Moreover, while important cortical involvement is predictive of a high risk of fracture, permeative lesions with ill-defined borders and heterogeneous pattern can also be related to a significant risk of fracture that is largely underestimated since they are more difficult to identify. Eventually, despite their sclerotic pattern, blastic or mixed lesions can also increase the risk of fracture [45].

**Figure 1:**
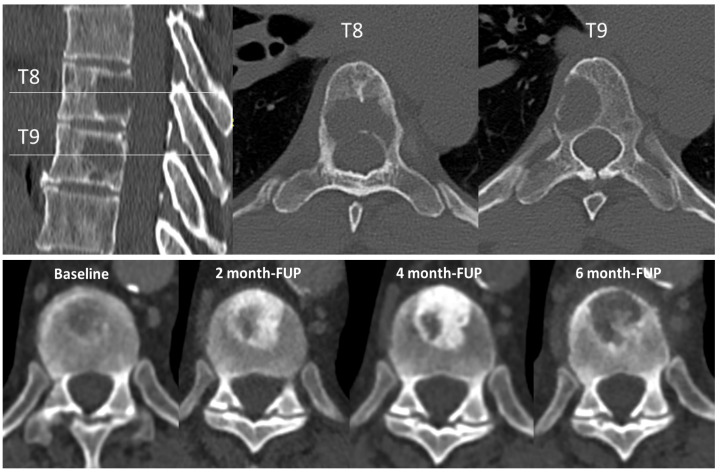
CT scan. Upper part: Multiplanar sagittal and axial reconstructions of the dorsal spine showing lytic lesions of myeloma involving T8 and T9 vertebrae. Lower part from left to right: progression of the size and condensation of a urothelial cancer bone metastasis under immunotherapy (avelumab), with initial lytic aspect and partial sclerosis of the lesion at 2 months. At 4 months, the sclerosis increases except in a focal region suspected of local progression. Confirmation of local progression with lysis of the sclerotic matrix at 6 months. FUP, follow-up.

Computed tomography (CT scan)—CT scan provides a better morphological evaluation of the cortical and trabecular bone than plain radiographs, and constitutes the preferred method for assessing the risk of fracture, facilitated by multiplanar or 3D reconstructions (Figure 1—upper part). The oncological follow-up regularly requires CT scans and includes the evaluation of the axial skeleton. Three-dimensional reconstructions of the axial skeleton should become the gold standard to correctly follow the fracture risk changes in addition to the tumor response. It is all the more important that with hormonotherapy, targeted therapies, and immune checkpoint inhibitors, bone tumor lesion phenotype may progress within a short period of time from a massive osteolytic lesion with a high fracture risk to a dense in-remission lesion with a low fracture risk [39]. Later, as the tumor re-progresses, bone tumor lesion may also progress and again weaken the bone, increasing the fracture risk. This is illustrated by chronological CT images of a vertebrae presented in Figure 1—lower part. CT scans of the limbs will be performed upon clinical or radiographic signs. It avoids superimpositions, increasing the sensitivity for osteolytic, blastic, and mixed lesions. Thus, advanced bone lesions are easily followed using CT, and comparison with previous data allows assessing of local progression.

However, bone metastases from solid tumors are not eligible yet to target lesions in RECIST 1.1 [46]. Pseudo-progression related to immunotherapy have been reported with mainly sclerotic changes but the underlying mechanism is still debated [47,48]. Early bone marrow invasion does not result in bone lysis but in a simple increase in density. Recent advances in dual-energy scanners have facilitated the detection of bone metastasis [49], but an association with fracture risk has not yet been clearly reported.

Magnetic resonance imaging (MRI)—MRI is a technique sensitive to bone marrow infiltration and has a better sensitivity for detecting small lesions before they result in bone destruction detectable with CT or radiography. The number and spreading of lesions and the local infiltration constitute indirect estimates of bone fragility. Nevertheless, MRI is less efficient for the characterization of bone destruction compared to CT. FEA-based modeling has been reported ex vivo on vertebrae or in vivo using 15 min acquisition time sequences, which is hardly feasible in a clinical MRI-based routine [50]. Low-TE or synthetic sequences allow good depiction of cortical bone and could be an alternative approach to CT [51]. They have been tested for the detection of bone metastasis [52], but the assessment of risk fracture with these sequences has not been reported yet.

MRI also offers a good option to show local bone edema related to a pathological fracture that may occur on a bone weakened by metastases. However, discriminating small fractures from local progression of the lesion can be difficult using MRI. The detection of subtle impaction of the trabecular bone or subtle cortical fracture is easier on T1 weighted images or on high-resolution sequences, and often benefits from the combination of a CT scanner assessment to better depict the structural changes of the bone.

Finally, it is important for physicians, surgeons, and radiotherapists to take into account that axial skeleton MRI detects epiduritis extension of the tumor. We clearly note here that MRI and CT-scan provide complementary information, explaining why they are both requested in bone tumor consultation meetings to decide the strategy of complex bone metastasis care.

Nuclear medicine—As previously mentioned, bone scan reveals the remodeling of the bone in reaction to the metastases and has a greater sensitivity than radiographs. The association of bone scan and regular CT has greatly increased its specificity but also its sensitivity, however remaining inferior to PET-CT or whole-body MRI in most lytic bone metastasis contexts. Moreover, all these nuclear medicine techniques are quite sensitive to the occurrence of small pathological fractures. The lack of specificity of bone scan alone has been almost compensated for by the association with a cross-sectional imaging modality.

### 4.3. Bone Metastatic Fracture Risk Scores and Their Limit

Long bones—The Mirels’ score [53] has been developed for helping physicians to predict the fracture risk of long bones (femur) and deciding the type of intervention to be performed between orthopedic surgery and radiation therapy. This score is currently the gold standard for long bones, and is only based on X-rays. The Mirels’ score relies on four parameters to identify high fracture risk lesions requiring surgery, each scored from 1 to 3 points: site, pain, size, and type of lesions. The size of the cortical defect is certainly the main parameter for long bones. However, the Mirels’ score lacks sensitivity and specificity [54,55,56,57,58], especially in intermediate situations [54]. Thus, there is a need to obtain an accurate evaluation of the strength of the tumoral bone segment, which will provide physicians with a more accurate tool to optimize locomotor strategy and oncology programs, to prevent bone fractures, and improve the survival and quality of life of these patients.

Spine—Vertebrae are highly vascularized and are also often affected by metastases, especially in the posterior part of the vertebral body [59]. Bone metastases located in vertebrae may be responsible for severe pain, immobilization, and reduced quality of life. However, the main issues of vertebral metastases lie in the risk of pathologic fracture (10–30% of all cancer patients) and spinal cord compression (5%) [60], which may lead to paraplegia. In 2010, the SOSG (Spinal Oncology Study Group) introduced the spinal instability neoplastic score (SINS) as a way of standardizing the categorization of spine lesions caused by neoplastic disease [61]. The criteria are based on input from a panel of 30 spine surgeons and include six components: tumor location, pain characteristics (pain-free, mechanical, or non-mechanical pain), type of bone lesion (lytic, blastic, or mixed), presence of radiographic spine alignment, degree of vertebral body collapse, and posterolateral involvement. Each of these components is given a score and summed to generate a final SINS score ranging from 0 to 18. Lesions scoring 0–6 are categorized as stable, 7–12 as potentially unstable, and 13–18 as unstable, with surgical advice recommended for lesions scoring ≥7. Since the introduction of the SINS score, studies have showed the reliability of this score [62,63] but there is limited clinical data from patient cohorts on its ability to predict fracture after conventional radiation therapy [64]. A recent meta-analysis was published on the accuracy and precision of the SINS score for predicting vertebral compression fractures after radiotherapy in spinal metastases [65]. Although this tool correctly predicts extreme cases [61], it remains unsatisfactory for intermediate modes (score between 7 and 12) [64]. In such situations, it remains difficult for physicians to choose the adequate treatment as there is no clear guideline. The Tokuashi score published in 2005 [66] is another score adopting a more overall approach using the performance status, motor deficit, and number of bone and soft metastases. Because the Tokuashi score is old and predates the molecular and immunotherapy era, it takes into account the primary lesion before the genetic information and molecular patterning of the tumors or the PDL1 expression. Thus, the overall perspective and survival is altered, making this score obsolete.

## 5. Emerging Tools

As long as patient prognosis exceeds 3–6 months, metastatic lesions with a high fracture risk are surgically treated using prophylactic osteosynthesis or prosthetic replacement, whereas low-risk lesions are treated conservatively using radiotherapy, chemotherapy, hormonal therapy, cementoplasty, or bisphosphonates [67]. However, it is difficult to discriminate between low- and high-risk lesions based on the available radiographic imaging material, even for experienced physicians [68,69,70,71]. We have previously seen that the risk of fracture remains poorly estimated despite the availability of fracture risk scores in intermediate clinical situations. Thus, there is a need to develop innovative tools to better predict the bone metastatic fracture risk.

### 5.1. Key Concept of Biomechanics and Numerical Simulation

**Figure 2:**
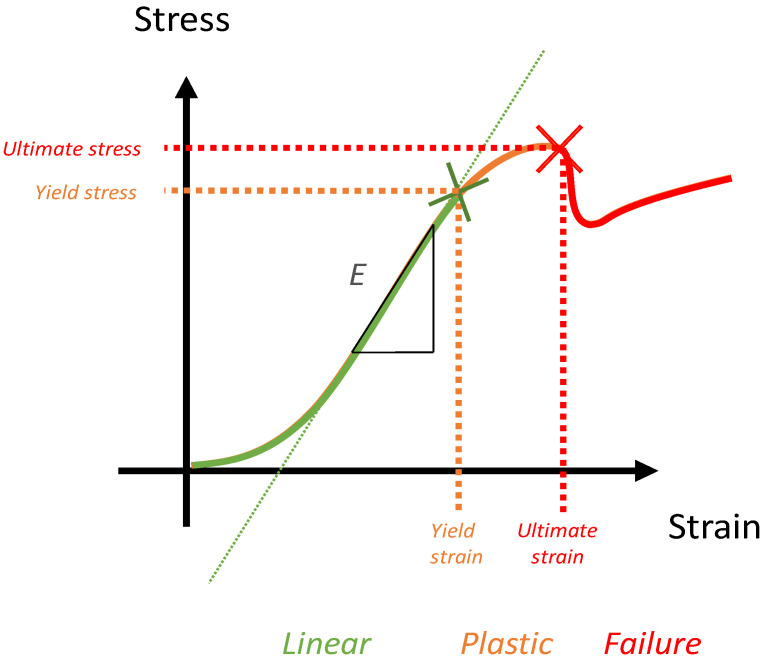
Biomechanics. An example of an idealized mechanical test curve. A load-displacement curve is transformed into a stress–strain curve with geometric parameters. The green part is the linear part corresponding to the elastic domain. The orange part corresponds to the plastic deformation domain. The ultimate stress (in red) corresponds to the failure but can only be numerically determined using a failure criterion.

Numerical simulation is one of those tools. Finite element (FE) models and simulations are used to generate detailed distributions of stress and strain in bones and are essential for understanding their mechanical behavior. These models are built on real clinical images and are called “patient-specific” modelling. Parameters reported from these models are mostly the bone strength or ultimate stress (Figure 2) and the final aim is to obtain a fracture risk assessment. The purpose is to obtain a reliable numerical simulation, able to reproduce experimental biomechanics with firstly, the elastic phase, and secondly, the plastic phase up to the breaking load (Figure 2).

**Figure 3:**
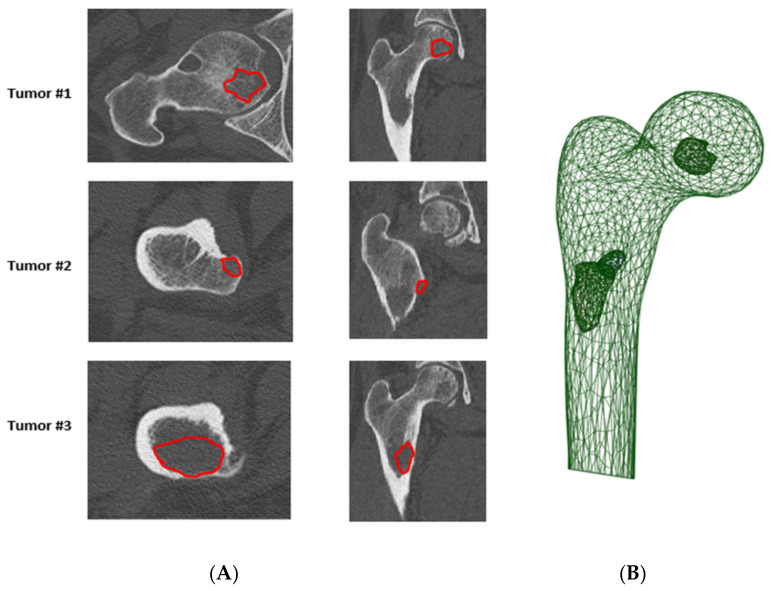
(**A**) Manual contour (in red) of a tumor, performed by a radiologist (annotator), (**B**) Patient-specific finite element model of proximal femur, with a specific representation of the metastasis.

Geometry is provided by image acquisition and transformed into a mesh (model). To solve mathematical equations and obtain displacements and loads, a discretization of the geometry is done into small elements. The grey levels of the images are converted into density [72] and then into intrinsic material properties (mainly Young’s modulus; Figure 3) [73]. Numerical simulation has been set for osteoporosis and has become reliable [74,75]. For bone metastases, scientists and engineers have major issues to contend with. Indeed, the attention to mesh quality [76], the specific material properties in model validation, the appropriate energy balance methods, and the reporting of these metrics have not kept pace with the general use of finite element modeling [77,78]. Simulation or analysis involves applying different loading conditions on the model with specific boundary conditions and failure criteria to estimate the bone strength. Special care must therefore be taken to obtain valid numeric models [79,80]. This area of research is under active development.

### 5.2. Femoral Fracture Risk Assessment Using Numerical Simulation

The strength of the metastatic bone depends on the characteristics of both the bone and the lesion, i.e., for the bone, size, shape, three-dimensional variation in density of trabecular and cortical bone, and bone microarchitecture, and for the lesions, size, shape, and type [81]. Next, the risk of hip fracture due to metastatic lesions depends on (1) the strength of the proximal femur and (2) the forces applied to the femur. Many of these quantitative parameters can be provided by a routine CT scan. However, although most patients at risk of bone metastatic lesion undergo CT to visualize the size and location of the metastases, quantitative parameters are currently not used. Only a qualitative evaluation is routinely performed for the interpretation of CT and standard X-ray. We believe that taking advantage of quantitative data from these CT scans would be very useful to improve fracture risk prediction. In a recent study, Benca et al. [81] showed that the lesion site has a large impact on the magnitude of the reduction in biomechanical properties. Nevertheless, CT parameters alone are not sufficient to assess femoral strength. Subject-specific models would be a powerful tool for predicting fracture risk in patients with bone metastases. For example, in the context of osteoporosis, outstanding progresses have been achieved in bone strength evaluation and fracture prediction using numerical simulation and finite element analysis [82,83,84].

A few studies have attempted to transfer this technology in oncology settings, through the development of FE models of ex vivo femurs with a simulated metastatic lesion [67,68,70,85,86,87,88,89] and metastatic femurs of patients [71,90,91,92,93,94,95]. As previously mentioned, FE models and simulations are used to generate detailed distributions of stress and strain in bones and are essential for understanding their mechanical behavior. Studies have shown that FE simulations can (1) accurately predict the experimental failure load of the femur with a simplified simulated lesion when single-limb stance loading conditions are applied [67,96], and (2) outweigh the performance of clinical experts when applied to patients, but still lack accuracy [70]. These first studies demonstrate that patient-specific FE analyses constitute a promising tool for the prediction of fracture risk in metastatic bone disease [56,90,97,98]. Up to now, the accuracy of these models and the ability to implement them in clinical practice was hampered by several limitations. First, except for Johnson et al. [89], the available models consider a single-limb stance loading for assessing the risk of fracture related to metastatic disease. Fractures of metastatic femur usually occur spontaneously during daily life activities such as walking, sit to stand, turning, rising; therefore, different loading conditions need to be developed and incorporated into the simulation to predict the fracture risk with higher accuracy. Second, most of the models provide an overall criterion associated with the fracture risk. A recent study using animal models showed that a local analysis performed around the tumor results in a better failure prediction compared to a general analysis [99,100,101,102], suggesting that a local failure criterion could further improve the prediction of fracture and the location of the failure. Third, knowledge of the structural and mechanical properties of the tumor and the surrounding bone remains limited [103]. The material properties used in FE modelling are generally based on empirical studies investigating the relationship between CT intensity and material behavior of the healthy bone tissue. Yet, the composition, and consequently material behavior, of the bone metastatic tissue may be pretty different from the healthy tissue and influence the bone strength [104]. Researchers should tackle these different aspects in order to improve the sensitivity and specificity of fracture risk prediction in oncology.

### 5.3. Vertebral Fracture Risk Assessment Using Numerical Simulation

To overcome SINS limitations, FE models are also in development to evaluate the strength of vertebrae [105,106,107]. Only a few studies have presented experimentally-validated FE models for strength assessment of vertebrae with defects [104,108], and several issues remain to be addressed. The experimental studies investigating the influence of the defect size on the vertebral strength have drawn conflicting results. In some cases, defect size was considered as a predictive factor of vertebral strength [109,110,111], while the opposite has been reported in other studies [112,113,114]. Hence, defect size is not the only predictive factor for vertebral strength reduction. Defect location is also predictive, especially for vertebral segments including the pedicles or costovertebral joints [112,113,115,116,117]. A previous study also showed that transcortical defects reduce the vertebral strength [116], meaning that defect type is also a factor to be considered. In published studies, defects were mostly created by drilling to mimic lytic metastases, which led to bone destruction. However, this technique caused cortical damage [107,110,112,113,116,117,118,119], which lead to vertebral strength reduction [116]. Another approach is to directly reuse experimental compression protocols developed in osteoporosis contexts in intact vertebral bodies for inducing anterior wedge-shape fracture [120] and test metastatic vertebrae. This strategy did provide promising results [104]. More recently, in a translational approach, another group has presented a feasibility study to assess the mechanical weakness of vertebrae affected by primary tumors [121]. They observed that the size and location of the lytic lesion are relevant in driving the spinal biomechanical instability [122]

### 5.4. Tools to Assess Loadings Applied to Metastatic Bones

To predict the risk of fracture from a mechanical point of view, in addition to the bone strength that could be assessed using a patient-specific FE model, the loadings applied to the bone should be defined and taken into account. This is a crucial issue. In case of bone metastases, the loadings of interest are those that reflect daily life movements. Since qCT are performed in the lying position, that represents a limitation of the previously mentioned models.

**Figure 4:**
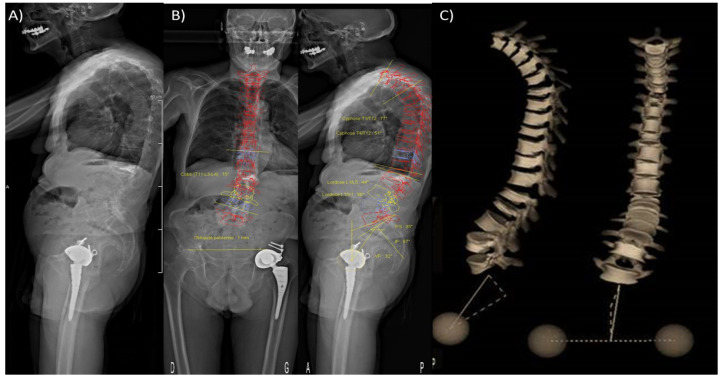
(**A**) Low dose full-body scan in standing position obtained using the EOS system^®^. (**B**) Semi-automatic measurement of spinal statics and pelvic angles on front and profile images. (**C**) 3D-reconstruction of the whole vertebral column in standing position.

Static loadings due to the sagittal imbalance could be assessed from medical imaging. In particular, the low-dose EOS system [123] allows obtaining full-body radiographs in standing position to assess loadings on the spine and on specific vertebrae [124]. (Figure 4.)

The EOS system, in comparison to standard radiographs, presents the advantages of low radiation, high-quality image [125], and potentiality to build a patient-specific FE model to predict bone strength [105,126]. In the case of a vertebra without metastasis, a sensitivity study has previously shown that a 1-cm anterior displacement of the loading on a vertebra would approximately decrease the vertebral strength by 50%. This highlights the influence of the postural alignment on the risk of fracture [124], and joins the observation of the highly increased and early incidence of vertebral fracture risk following a first fracture and the classic “vertebral fracture cascade”. This also highlights the importance of preventing the first fracture.

Dynamic loadings (e.g., along a gait cycle) can be measured in vivo using instrumented implants [127]. However, very few data are available. Musculoskeletal models [128] have been developed to assess contact forces in a joint (e.g., the hip and the knee) and the forces in each muscle during locomotion and exercise. The current challenge for these models lies in their validation [129], as no direct measurement of the forces in the muscles can be performed in vivo. Up to now, no data has been reported in bone metastatic settings. This is an important challenge for the coming years to guide patients in their rehabilitation once cancer stabilization is obtained.

Knowing the loadings in specific daily activities would be of interest to define the range of variations of the loadings applied to metastatic bones. This knowledge could allow the prediction of probabilistic results instead of current deterministic values. Such models would include the uncertainties due to the prediction of bone strength and the uncertainties due to bone loadings.

## 6. Conclusions

**Figure 5:**
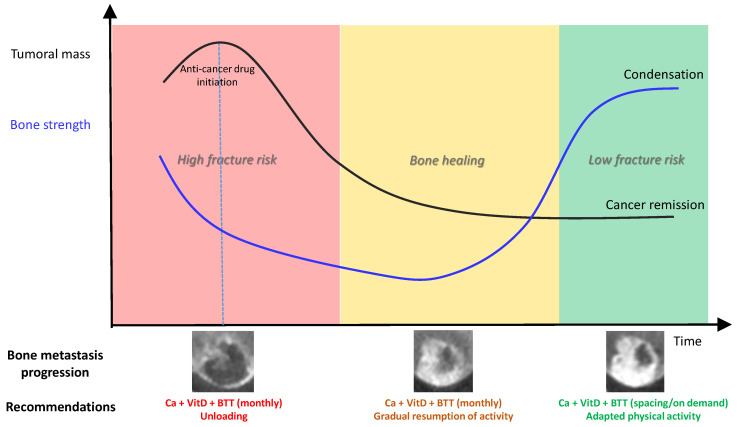
Conceptual model of bone strength progression according to osteolytic bone metastasis response to anti-cancer drug. BTT, bone-targeted treatments.

The bone metastasis field has entered a new clinical era. Thanks to therapeutic progress, patients live longer, reaching stabilization and prolonged remission in many cases. The high fracture risk of a bone metastasis becomes a changing and temporary condition for which multidisciplinary teams should aim at preserving locomotion by preventing pathologic fracture and treat pain. These clinical situations highlight the crucial need to develop personalized and reliable fracture risk evaluation and its follow-up to guide the locomotive strategy (Figure 5). This will also guide the physical activity advised to patients. qCT-based numerical simulation and artificial intelligence constitute promising tools. They require urgent clinical validation and consensus from biomechanics research teams to allow the transfer into routine clinical practice.
